# Importance of Global Genomic Data Sharing to Prevent Illnesses Linked to Commercially Distributed Foods

**DOI:** 10.14252/foodsafetyfscj.D-25-00003

**Published:** 2025-09-26

**Authors:** Matthew E. Wise, Christine C. Lee, Laura Gieraltowski, Thai-An Nguyen, Megin Nichols, Morgan N. Schroeder, Heather A. Carleton

**Affiliations:** Division of Foodborne, Waterborne, and Environmental Diseases, U.S. Centers for Disease Control and Prevention, 1600 Clifton Road, Atlanta, GA, 30329

**Keywords:** data sharing, foodborne disease, whole genome sequencing

## Abstract

Changes in food production and distribution, including global distribution of foods, have affected the types of foodborne disease outbreaks that occur. As a result, outbreaks are frequently identified that cause illnesses affecting people across wide geographic regions. Pathogen subtyping is the most effective way to detect these outbreaks, by grouping together cases of foodborne illness that likely share a common source. Over the last decade, the use of whole genome sequencing-based subtyping methods for foodborne bacteria has rapidly expanded. These methods not only provide a much higher resolution view of the bacterial genome, but also open the door to improved sharing of genomic data internationally. We describe four recent outbreak investigations linked to globally distributed foods and other products in which international genomic data sharing played a key role in the investigation: a listeriosis outbreak linked to enoki mushrooms and three different salmonellosis outbreaks linked to onions, sesame paste, and small turtles. Although there are technical and cost barriers to expanding the use of whole genome sequencing and other high-resolution subtyping methods, advancements and accessibility in using these technologies are creating novel opportunities for widespread global implementation so that international collaborations can detect, investigate, and stop outbreaks and reduce the impact of foodborne illnesses globally.

## Introduction

As food production and distribution have evolved over the last several decades, the types of foodborne disease outbreaks and patterns of foodborne illness that occur have similarly evolved. In the United States, the number of outbreaks detected and investigated that simultaneously affect people across a wide geographic distribution has increased^1^^)^. These outbreaks are commonly linked to commercial distribution of a food item that was contaminated during growing, processing, or production. Globalization of food systems has resulted in an increase in outbreaks that not only cross jurisdictional boundaries within countries, but also extend across national borders^2^^)^. Robust processes for international collaboration and data sharing will be needed to rapidly investigate these international foodborne outbreaks.

Central to identification and investigation of these highly disseminated foodborne outbreaks is the ability to group together illnesses that are likely to share a common food source. This has been achieved through progressively higher-resolution methods to determine how similar pathogens are to one another. Serotyping is an early example of a type of characterization that allows more similar pathogens to be grouped together. Subtyping methods evolved beyond serotyping to techniques such as pulsed-field gel electrophoresis (PFGE), which allowed investigators to make more accurate inferences about the genetic similarity of organisms. In fact, PFGE has been a common method used across many countries to subtype bacterial foodborne pathogens and to identify outbreaks. While PFGE has proved highly effective at identifying disseminated outbreaks, especially within countries, it did come with limitations. The first was that for clonal lineages of pathogens, PFGE did not always have the discriminatory power to partition disease-causing organisms into groups that were likely to share a common source. Another major limitation was that it could be challenging to share PFGE information between countries. In particular, the use of different PFGE protocols that might rely on the use of different restriction enzymes, gel run settings, analysis software, or strain nomenclature was a significant barrier to sharing subtyping data internationally, especially in real-time for public health outbreak response activities.

Since 1996, PulseNet served as the U.S. surveillance system for identifying and subtyping enteric pathogens that cause foodborne illness. Public health laboratories used standardized PFGE protocols to submit data from clinical isolates for surveillance and outbreak detection coordinated by PulseNet^3^^)^. The increased accessibility and affordability of whole genome sequencing (WGS) and the parallel development of bioinformatic tools for analyses created an opportunity to address the limitations of PFGE and improved characterization of enteric pathogens. In 2019, PulseNet transitioned to using WGS data for foodborne pathogen surveillance^4^^)^. WGS enables a consolidated laboratory workflow for species identification, toxin profiling, serotyping, and antimicrobial resistance determinant predictions. Furthermore, standardization of laboratory protocols and the establishment of minimum quality control metrics for WGS data tailored for enteric pathogen characterization^5^^)^ created a foundation for comparing sequences generated not only within the PulseNet network but also from around the world. Bioinformatic approaches like high-quality single nucleotide polymorphism (hq-SNP)^6^^)^ and multilocus sequence typing (MLST) schemes improved cluster detection of closely related sequences and better discrimination from outgroups that have neither genomic nor epidemiologic linkages compared to conventional techniques like serotyping alone^7^^)^. Therefore, the standardization of WGS workflows and quality thresholds created an opportunity to classify pathogens so that isolates from human, food, animal, and environmental samples can be compared side-by-side for improved outbreak detection and investigation.

Storage of high-throughput sequencing data is hosted by and mutually shared among the International Nucleotide Sequence Database Collection (INSDC), which includes the National Center for Biotechnology Information Sequence Read Archive (SRA), the European Bioinformatics Institute (EBI), and the DNA Database of Japan (DDBJ). Sequence data from PulseNet, the U.S. Food and Drug Administration (FDA) GenomeTrakr, the United States Department of Agriculture, and other agencies are also shared through the SRA, which facilitates monitoring outbreaks of foodborne pathogens in the United States^8^^)^ and globally. Investments in global laboratory capacity building have accelerated the implementation of WGS. PulseNet International is a global and regional laboratory network that facilitates genotyping-based foodborne pathogen surveillance in more than 100 countries across eight regions around the world through laboratory capacity building, technical assistance, and real-time information and data sharing. The barriers for maintaining WGS-based surveillance vary by region, especially in low and middle-income countries but primarily include training and expertise gaps and the need for analysis tools^9^^)^. To this end, PulseNet International recently invested in meeting these needs through laboratory trainings to build sequencing capacity in the Middle East, Asia Pacific, and Africa^10^^)^ regions. Publicly available platforms with easily navigable interfaces allow users with limited bioinformatics experience to submit sequence data for species identification, subtyping, and comparisons with other sequences uploaded from users around the world. These include EnteroBase coordinated by The University of Warwick^11^^)^, Pathogenwatch^12^^)^ coordinated by the Wellcome Sanger Institute, and PubMLST^13^^)^ which is a collection of open-access databases for molecular typing. Taken together, these tools and data sharing platforms are enhancing the quality of foodborne disease surveillance internationally and provide the foundational infrastructure for efficient cross-national sharing of genomic data for foodborne pathogens.

## Genomic Data Sharing in Action

Increasing adoption of WGS-based subtyping methods has already led to numerous examples in which global sharing of both genomic and epidemiologic information has been critical to determining the source of outbreaks and taking public health action to prevent additional illness. Below, we highlight four examples of outbreak investigations that relied on international data sharing and collaboration and illustrate the potential benefit of further investment in these activities.

### Multinational Listeriosis Outbreak Linked to Enoki Mushrooms

In 2017, the U.S. Centers for Disease Control and Prevention (CDC) worked with state health departments to investigate two separate clusters of *Listeria monocytogenes* infections, but both investigations were closed without identifying a food source^14^^,^^15^^)^. In 2018, identification of new illnesses with isolates closely related to one of these clusters prompted CDC to re-open its investigation and to eventually combine the two initial 2017 clusters into a single epidemiologic investigation. Unfortunately, the combined 2018 investigation also did not identify a source for the infections. In 2020, the Canadian Food Inspection Agency (CFIA) uploaded WGS data to the U.S. NCBI database for *Listeria* isolated from a domestic mushroom from Canada which was closely related genetically to isolates from the U.S. illnesses. This was followed by CFIA uploading WGS data for *Listeria* isolated from enoki mushrooms imported into Canada that matched the outbreak strain. After the Public Health Agency of Canada (PHAC) identified six historical clinical isolates matching the outbreak strain, U.S. and Canadian investigators opened a collaborative outbreak investigation. In the United States, state health departments collected samples of enoki mushrooms from stores reported by ill people in the outbreak, which yielded the outbreak strain of *Listeria*. Inquiries to the European surveillance portal for infectious diseases (EpiPulse) uncovered that French authorities had identified the same strain of *Listeria* in enoki mushrooms imported from South Korea. Additional import sampling by both the U.S. FDA and Canada in 2020 identified the outbreak strain in enoki mushrooms imported from South Korea. Illnesses with the strain were also confirmed by Australia. The multinational sharing of genomic and epidemiologic data in this outbreak led to enoki mushroom recalls in the United States and Canada, ending a years-long outbreak and preventing additional illnesses.

### Salmonellosis Outbreak in the U.S. and Canada Linked to Onions

In July 2020, CDC PulseNet identified a cluster of ten *Salmonella* Newport illnesses with isolates that were closely related by WGS^16^^)^. At the same time, PHAC was notified by two Canadian provinces of increases in *Salmonella* illness that were confirmed to be caused by *Salmonella* Newport that was closely related genetically to isolates from the U.S. illnesses^17^^)^. The United States and Canada initiated a multinational investigation into the source. Both countries initially gathered food history information from ill people from hypothesis generating or routine enteric disease questionnaires administered by local jurisdictions, which revealed a high proportion of ill people eating onions prior to illness onset. Epidemiologic information gathering then transitioned to more focused questionnaires that included questions on onions and other produce as well as identification of illness sub-clusters. Illness sub-clusters are defined as when two or more unrelated ill people included in an outbreak dine at a common restaurant, shop at a common grocery store, or eat food at a common event prior to becoming ill. Sub-clusters are important in widespread outbreaks linked to commercially distributed foods because they indicate that the contaminated ingredient was sold or served at the sub-cluster. Both the United States and Canada identified numerous illness sub-clusters in this outbreak. U.S. sub-clusters were commonly identified at Mexican-style restaurants where many dishes included onions, tomatoes, and cilantro. The overlap of these ingredients across numerous dishes made it difficult to implicate a single ingredient as the source. However, in Canada illness subclusters were identified at restaurants serving other cuisine types and at congregate living settings. The lack of tomato and cilantro exposure in many of the Canada sub-clusters helped the United States also narrow its investigation focus to onions. Traceback of onions consumed by ill people at illness sub-clusters in Canada by CFIA and in the United States by FDA implicated a common grower of onions in California as the source of the outbreak, leading to several recalls. Without genomic data establishing a connection between illnesses in the United States and Canada and sharing of epidemiologic and traceback information between the two countries, implicating onions as the source – particularly in the United States – would have been significantly more challenging.

### Four Salmonellosis Outbreaks Linked to Contact with Small Turtles

Although this review focuses on outbreaks linked to commercial distribution of contaminated foods, highly disseminated outbreaks are also commonly linked to commercial distribution of companion animals or pets, such as backyard poultry or reptiles. In 2015 the FDA contacted CDC after receiving a report about a *Salmonella* illness in a child linked to contact with a small pet turtle (shell length <4 inches), a recurring source of salmonellosis outbreaks in the United States^18^^)^. This prompted CDC to review the PulseNet USA database for any *Salmonella* isolates with the same PFGE pattern, as well as recent illnesses with any *Salmonella* strains previously linked to contact with turtles. Four different outbreaks were identified, including two serotype San Diego outbreaks, one serotype Poona outbreak, and one polyclonal outbreak that included serotypes Poona and Pomona. Both PulseNet International and EpiPulse, were queried for illnesses with the same PFGE patterns. Whole genome sequencing was performed to further characterize the relatedness of isolates that were indistinguishable by PFGE. International queries for related illnesses led to identification of four illnesses in Luxembourg (*Salmonella* Poona) and one in Chile (*Salmonella* Pomona) and exchange of WGS data confirmed these as being related to isolates from the U.S. outbreaks. The origin of turtles was traced to U.S. turtle farms in Louisiana. CDC informed the World Health Organization about the outbreak, highlighting the link between Louisiana turtles and illnesses in the United States, Europe, and South America.

### Multi-country, Multi-serotype Salmonellosis Outbreak Linked to Sesame Paste

This investigation began in April 2021 after health officials in Sweden identified clusters of *Salmonella* Havana and *Salmonella* Mbandaka infections that were dispersed geographically in the country^19^^)^. After communication to other European countries through EpiPulse, four additional *Salmonella* serotypes (Amsterdam, Kintambo, Orion, and Senftenberg) were added to the investigation and WGS case definitions were developed for European countries to include cases in the outbreak. WGS ultimately revealed there to be additional illnesses with the outbreak strains in Denmark, Germany, Norway, and The Netherlands. Genomic data were shared with the United States and Canada, which identified cases with the outbreak strains of Mbandaka (United States and Canada) and Havana and Orion (Canada only). Product testing in European countries identified numerous *Salmonella* serotypes (including outbreak strains) from sesame paste (tahini)-containing products imported from Syria and many ill people reported consuming sesame paste-containing foods prior to becoming ill. Illnesses with the outbreak strain were documented going back to 2019, suggesting contaminated products were available for sale (or in homes and restaurants) for an extended period, despite several recalls and other control measures beginning in 2020. In 2021, the European Food Standards Agency and the European Centre for Disease Prevention and Control issued a joint rapid outbreak assessment, highlighting the continued risk of salmonellosis from sesame-based products imported from Syria.

## Discussion

The four outbreak examples we present are a small subset of the numerous foodborne and animal contact outbreak investigations conducted around the globe that leverage WGS technologies, but they offer a strong illustration of how international data sharing can be of mutual benefit, leading to improved food safety across all countries involved in these investigations. Each of these examples was from an outbreak in which the United States had illnesses, but we are aware of the existence of numerous multinational outbreak investigations without U.S. cases that have also benefited from sharing genomic data between countries. Rather than being an exhaustive review of multinational outbreak investigations, we believe these examples provide a robust picture of how genomic data sharing can play a role in supporting public health action during international outbreaks.

Although there are clear technical advantages to using WGS-based methods to characterize enteric bacteria, there are challenges in adopting and using WGS routinely. First, while the cost to sequence an isolate has dropped dramatically, it is still more expensive than other subtyping methods. This is due to the initial investment in equipment and the ongoing procurement of reagents needed for WGS. Conventional subtyping methods (such as serotyping) have a significantly lower cost per sample and utilize inexpensive and commercially available reagents^20^^)^. Second, expertise in both generating and analyzing sequence data is needed and may require new skills and tools compared to past microbiologic methods. In particular, staff with bioinformatics expertise to generate the analyzed sequence results needed for identifying potential outbreaks are often limited in public health settings. Third, WGS generates large quantities of data that need to be both stored and transmitted; even constraints such as the internet bandwidth available to laboratories can be a barrier to implementation^9^^,^^21^^)^. Fourth, clinical settings are increasingly using culture-independent diagnostic tests (CIDT) for identification of enteric pathogens directly from primary specimens such as stool^22^^)^. These tests provide rapid results to the clinician but do not generate an isolate which is required for WGS. In the United States, many public health laboratories take on the task of isolating bacteria from specimens testing positive by CIDT, which can add cost and decrease timeliness of WGS. Fifth, sharing of WGS data between countries requires a level of trust and agreements in place, such as data use agreements, that outline how the data is shared and how it can be used. Setting clear expectations for permitted uses of shared data, including protections for patient privacy, are critically important for effective sharing of genomic data. Finally, standard quality metrics for WGS data and validated and interoperable bioinformatics workflows are also needed to ensure that different countries would identify the same isolates as coming from cases belonging to a particular outbreak.

The precision offered by WGS enables investigators to draw stronger associations between pathogens and their sources, even when faced with less conclusive epidemiologic and traceback evidence. By integrating genomic data with traditional epidemiologic and traceback information, public health investigators can weigh the evidence across all lines of inquiry more effectively, leading to more robust conclusions about outbreak origins and transmission pathways. This comprehensive approach not only enhances the overall investigation process but also allows for more efficient use of resources: smaller, more agile teams can leverage WGS to conduct thorough investigations without the need for extensive staffing or resource-intensive methods. As a result, public health agencies can respond more swiftly and effectively to enteric disease outbreaks while optimizing their operational capabilities.

Recent findings highlight the economic viability of WGS in pathogen identification and surveillance. Case studies from Europe and the Americas demonstrate that even preventing a small percentage of *Salmonella* cases or a single death can make WGS a cost-neutral investment^23^^)^, even considering the higher up-front costs for equipment, reagents, and bioinformatics infrastructure. The potential savings from reduced healthcare costs, minimized productivity losses due to illness, and fewer food recalls far outweigh the initial investment needed to establish WGS infrastructure. This positions WGS as an important tool for public health and a cost-effective strategy for improving disease surveillance and enhancing outbreak response.

The benefits of adopting WGS-based subtyping extend well beyond individual countries, offering a shared opportunity for enhanced public health outcomes on a global scale. In an era of an increasingly globalized food supply, the ability to share genomic data more easily between countries offers a chance to find, investigate, and solve global outbreaks that may have previously been difficult to detect and investigate using past subtyping methods. As we have already seen, these multinational investigations can lead to identification of novel food-pathogen pairs, such as the example of *Listeria* and enoki mushrooms, providing additional opportunities to better understand how pathogens move through our global food supply. By collaborating through shared genomic data platforms and investing in advanced technologies like WGS, we can enhance our collective ability to track and respond to enteric pathogens. This joint approach not only improves our understanding of how these pathogens spread, but also enables more effective outbreak detection, response, and control strategies. These efforts have the potential to significantly reduce the incidence of foodborne infections more broadly, fostering safer food systems and improving public health outcomes globally.
